# United States Civil Service Examination

**Published:** 1923-04

**Authors:** 


### UNITED STATES CIVIL SERVICE EXAMINATION

DENTIST

U. S. Veteran's Bureau .........$2,400 to $3,600 a year
U. S. Public Health Service..... 2,400 to 3,600 a year

SIXTH U. S. CIVIL SERVICE DISTRICT (OHIO, INDIANA AND KENTUCKY)

APPLICATIONS WILL BE RATED AS RECEIVED UNTIL FURTHER NOTICE

The United States Civil Service Commission announces an open competitive examination for Dentist. Vacancies in this and similar positions, in the Veterans' Bureau and in the Public Health Service, for full-time duty at the above salaries, and for part-time duty at salaries to be determined by the services rendered, will be filled from this examination, unless it is found in the interest of the service to fill any vacancy by reinstatement, transfer, or promotion.

*Range in Salary.*—The entrance salary within the range stated will depend upon the qualifications of the appointee as shown in the examination and the duty to which assigned.

*Bonus.*—Appointees to the Public Health Service at annual compensation of $2,500 or less, whose services are satisfactory, may be allowed the increase granted by Congress of $20 a month.

*Certification.*—In filling vacancies in these positions, certification will be made by states, except that whenever there are few or no eligibles in any state the district secretary may extend the field of certification to include a part or all of the civil service district.

*Citizenship and Sex.*—All citizens of the United States who meet the requirements, both men and women, may enter this examination; appointing officers, however, have the legal right to specify the sex desired in requesting certification of eligibles.

*On account of the needs of the service, applications will be received until further notice. Papers will be rated as received and certification made as the needs of the service require.*

*Duties.*—To perform dental operations, either under supervision or independently, according to the assignment.

*Subjects and Weights.*—Competitors will not be required to report for examination at any place, but will be rated on the following subjects, which will have the relative weights indicated:

| Subjects | Weights |
| --- | --- |
| 1. Education and training.............. | 30 |
| 2. Experience ......................... | 70 |
| Total ............................ | 100 |

*Basis of Ratings.*—Competitors will be rated upon the sworn statements in their applications and upon corroborative evidence.

*Prerequisite Requirements.*—Applicants must show that they have been graduated from a dental school of recognized standing, and that they have had at least two years' full-time active practice as dentist within the past five years. For trainees of the Veterans' Bureau who have received the required degree but have not had the two years' practice, the requirement as to practice shall be waived and they will be given a rating of 70 per cent. in Subject 2; but they may be certified only for positions in the Veterans' Bureau where the entrance salary does not exceed $2,400.

*Medical certificate.*—The medical certificate in the application form must be executed by a physician in the Federal service where practicable. Persons selected for appointment may also be required to submit to a physical examination before actually entering on duty.

*Age.*—Applicants must have reached their twenty-fifth but not their seventieth birthday on the date of making oath to the application. Should the appointing officer so request, certification will be made of eligibles who are within reasonable age limits, except in the case of persons entitled to preference because of military or naval service to whom only the retirement age limit of 70 years applies.

*Retirement.*—Classified employes who have reached the retirement age and have served fifteen years are entitled to retirement with an annuity. The retirement age for railway mail clerks is 62 years; for mechanics and postoffice clerks and carriers, 65 years; and for others, 70 years. A deduction of 2½ per cent. is made from the monthly salary to provide for this annuity which will be returned to persons leaving the service before retirement with 4 per cent. interest compounded annually.

*Photographs.*—Applicants must submit with their applications their unmounted photographs, taken within two years, with their names written thereon. Proofs or group photographs will not be accepted. Photographs will not be returned to applicants.

*Applications.*—Applicants should at once apply for Forms 1312 and 2383, stating the title of the examination desired, to the Secretary, U. S. Civil Service Board, at any place listed below, or to the Secretary, Sixth U. S. Civil Service District, Cincinnati, Ohio. Applications should be properly executed, *including the medical certificate,* but excluding the county officer's certificate and the vouchers and filed with the District Secretary at the address below without delay.

SECRETARY, SIXTH U. S. CIVIL SERVICE DISTRICT,
403 Government Building, Cincinnati, Ohio.

Issued: February 14, 1923.

*Ohio.*—Ashtabula, Athens, Canton, Chillicothe, Cleveland, Columbus, Dayton, East Liverpool, Ironton, Lima, Mansfield, Marietta, Portsmouth, Sandusky, Toledo, Youngstown, Zanesville.

*Indiana.*—Angola, Bloomington, Evansville, Fort Wayne, Hammond, Indianapolis, Jeffersonville, LaFayette, Marion, Muncie, Richmond, South Bend, Terre Haute, Valparaiso, Vincennes.

*Kentucky.*—Ashland, Bowling Green, Covington, Henderson, Hopkinsville, Lexington, Louisville, Middlesboro, Owensboro, Paducah, Somerset.

---

### THE DETROIT DENTAL MFG. CO.

The ever increasing demand for Kerr instruments and preparations made it necessary for us to build a larger plant. January 1, 1923, we moved into our new factory, thus placing ourselves in a better position to serve you.

We wish to assure you of our appreciation of your patronage and will ever increase our efforts to maintain the high standard quality of our products.

That we may be deserving of your continued good will and co-operation is our aim.

